# Case of Exsection of Half of Inferior Maxillary; Reproduction of Bone

**Published:** 1877-07

**Authors:** Robert A. McLean


### ARTICLE VII.

### *Case of Exsection of Half of Inferior Maxilla for Caries; Reproduction of Bone.*

Dr. Robert A. McLean, Adjunct to the Chair of Surgery, University of California, reports (*Western Lancet*, April, 1877,) the case of Lena T., aged four years, who had been troubled with an enlargement of the left lower jaw since January, 1876. In the following May, the mother of the child consulted Dr. W. H. Mays, who found upon examination that there was extensive caries of the jaw, and sent the case to Dr. McLean for operation.

The general health of the child seemed fair. She was ruddy and had a good appetite. The left side of the face was greatly enlarged. Immediately beneath the angle of the jaw there was an opening through which pus escaped. There was also an opening in the gums behind the last molar tooth. The teeth upon the affected side were irregular, and in an advanced stage of decay. The probe, when passed into the external opening, came in contact with denuded bone in all directions. The mother stated that she first noticed a slight enlargement in January, after one of the back teeth had been removed. Pain was complained of, and the swelling continued to increase until March, when the skin broke, below the angle of the jaw, and a large amount of pus escaped. The pain now became less severe, but the face continued to swell on that side.

On May 21st, with the assistance of Dr. Mays and others Dr. McLean removed the left half of the jaw. Chloroform having been administered, an incision was made down to the bone, commencing at a point an inch below the articular extremity, along the posterior border of the ramus to the angle, and then forward along the inferior border of the body of the bone, to a point an inch to the right of the symphysis menti. A vertical incision was then made in the median line of the chin, commencing above, at the lower border of the inferior lip, and passing downward to join the horizontal incision. The vessels having been ligated, the periosteum elevator was introduced into the anterior extremity of the wound, and, proceeding backward along the body and ramus of the jaws, the bone was easily cleared of its membranous investment. The periosteum had been so thoroughly separated by the effects of the disease, that no difficulty whatever was met with in disarticulating the bone. It slipped out of the periosteal pouch like a finger out of a glove. In order to preserve the contour of the chin and the lower line of teeth, Dr. McLean decided to remove the bone no further toward the symphysis than the second incisor tooth, although there was considerable carious bone extending an inch to the right of the median line. The bone was divided with the chain-saw, and removed. The tissues of the chin were next lifted from the remaining half of the jaw, which was scarcely scraped, an inch to the right of the symphysis. Several small scales of bone which were detached, were removed from this locality.

The edges of the incision were brought together, and retained with silver suture, except at the point of junction of the vertical and horizontal incisions, where a small opening was left, in which was placed a tent to admit of drainage from the denuded surface of the remaining bone. The water dressing was then applied.

Immediate union occurred, except at the place where the tent had been allowed to remain. A slight discharge continued for three weeks from this opening, and then the entire wound healed, including the opening behind the angle of the jaw.

Four months after the operation the deformity was so slight as scarcely to be noticed. The lines of union were almost entirely obliterated. The bone was reproduced to an extent sufficient to admit of perfect motion of the jaw and the performance of all the functions of the mouth. About this time she unfortunately contracted diphtheria, and died after an illness of thirty-six hours.—*Monthly Abstract of Med. Science.*

---

